# Middle East Respiratory Syndrome Coronavirus (MERS-CoV): Infection, Immunological Response, and Vaccine Development

**DOI:** 10.1155/2019/6491738

**Published:** 2019-04-07

**Authors:** Ayman Mubarak, Wael Alturaiki, Maged Gomaa Hemida

**Affiliations:** ^1^Department of Botany and Microbiology, College of Science, King Saud University, Saudi Arabia; ^2^Department of Medical Laboratory Sciences, College of Applied Medical Sciences, Majmaah University, Majmaah 11952, Saudi Arabia; ^3^Department of Microbiology and Parasitology, College of Veterinary Medicine, King Faisal University, Saudi Arabia; ^4^Department of Virology, Faculty of Veterinary Medicine, Kafresheikh University, Egypt

## Abstract

Middle East respiratory syndrome coronavirus (MERS-CoV) first emerged in late 2012. Since its emergence, a total of 2279 patients from 27 countries have been infected across the globe according to a World Health Organization (WHO) report (Feb. 12th, 2019). Approximately 806 patients have died. The virus uses its spike proteins as adhesive factors that are proinflammatory for host entry through a specific receptor called dipeptidyl peptidase-4 (DPP4). This receptor is considered a key factor in the signaling and activation of the acquired and innate immune responses in infected patients. Using potent antigens in combination with strong adjuvants may effectively trigger the activation of specific MERS-CoV cellular responses as well as the production of neutralizing antibodies. Unfortunately, to date, there is no effective approved treatment or vaccine for MERS-CoV. Thus, there are urgent needs for the development of novel MERS-CoV therapies as well as vaccines to help minimize the spread of the virus from infected patients, thereby mitigating the risk of any potential pandemics. Our main goals are to highlight and describe the current knowledge of both the innate and adaptive immune responses to MERS-CoV and the current state of MERS-CoV vaccine development. We believe this study will increase our understanding of the mechanisms that enhance the MERS-CoV immune response and subsequently contribute to the control of MERS-CoV infections.

## 1. Introduction

Middle East respiratory syndrome coronavirus (MERS-CoV) is a novel human coronavirus that was previously called “novel human coronavirus Erasmus Medical Center” (HCoV-EMC). The virus was discovered for the first time in Saudi Arabia in 2012 by Zaki et al. [1]. The World Health Organization (WHO) has confirmed 2279 cases of human infections with MERS-CoV in 27 countries since 2012; 806 (35%) infected patients have died as of Feb. 13, 2019. However, Saudi Arabia still has the highest reported MERS-CoV mortality rate. Approximately 80% of the cases have been reported to occur there [2]. MERS-CoV belongs to the family *Coronaviridae*, order *Nidovirales*. It is one of the recently reported zoonotic viruses. The family *Coronaviridae* is classified into four genera (*α*, *β*, *γ*, and *δ*). Each genus is divided into linage subgroups. MERS-CoV belongs to lineage-C of the *β* coronaviruses [3, 4]. Although bats are the main reservoir for most coronaviruses, dromedary camels are considered the only known reservoir for MERS-CoV to date. Additionally, MERS-CoV isolated from dromedary camels is relatively closely related to some bat coronaviruses [5–7]. According to the WHO, MERS-CoV transmission between humans is possible and occurs in Middle East countries and the Republic of Korea [2]. Viral spread has been observed among health-care workers and among individuals visiting MERS-CoV-positive patients. The control of some of these outbreaks has been achieved by the local center of disease control and prevention (CDC) [2]. Immunocompromised individuals as well as patients with comorbidities are the groups most prone to severe MERS-CoV infection, which may lead to death of these infected patients in many cases [8–10].

Three MERS-CoV proteins are expressed on the envelope of the virus: the surface spike protein (S), the membrane glycoprotein (M), and the envelope protein (E). The S protein is responsible for viral entry via attachment to and fusion with the host cell membrane. MERS-CoV host cell receptors were identified to be cluster of differentiation 26, also known as dipeptidyl peptidase-4 [11, 12]. The interaction of MERS-CoV S proteins with the DPP4 receptor not only facilitates viral access into the host cell but also triggers signals that induce the immunosuppression of infected patients, enabling viral replication and spread [13].

Despite ongoing research on the development of specific therapies or vaccines against MERS-CoV, there is currently no effective prophylaxis or therapy for MERS-CoV, which hinders the treatment or control of the viral infection. Understanding the mechanism of the immune response against MERS-CoV infection will make the development of effective vaccine candidates achievable, especially if the vaccine candidates are strong enhancers for both cellular and humoral immunity. In this review, we will discuss how innate immunity and acquired immunity respond to MERS-CoV infections in light of the most up-to-date literature in this field of research. Moreover, we highlight the most recent advances in the field of MERS-CoV vaccines

## 2. MERS-CoV Innate Immunity

Dendritic cells (DCs) are important contributors to innate immunity and can trigger the production of large quantities of cytokines and chemokines. These cells have the ability to migrate from peripheral tissues to the lymphoid tissue to activate the T cell population [14]. Thus, DCs are considered potential targets for pathogen invasion, as they form bridges between innate and adaptive immunity [14]. Subsequently, both the T cell (cell-mediated immunity) and the B cell (humoral immunity) arms of the adaptive immune response are stimulated for a specific response [14]. The mechanisms of the immune response triggered by MERS-CoV infection and immune evasion strategies have not yet been fully studied. Interestingly, MERS-CoV has evolved strategies to manipulate innate immunity and prevent or block IFN production pathways [15]. This ability may contribute substantially to the high case-fatality rates of MERS-CoV-infected patients, especially those who are immunocompromised [9].

Pattern recognition receptors (PRRs), such as Toll-like receptors (TLRs) and retinoic acid inducible gene-I- (RIG-I-) like receptors, are essential mediators of the innate immune response [16–18]. After viral recognition by the TLR, one of the two different adaptor molecules is recruited—either MyD88 (myeloid differentiation primary response 88) or Toll/interleukin-1 receptor- (TIR-) domain-containing adapter-inducing interferon-*β* (TRIF). These molecules further activate the MAPK and NF-*κ*B pathways that are responsible for promoting the production of proinflammatory cytokines and IFNs [19–21]. Meanwhile, the spike protein of MERS-CoV triggers the expression of some negative regulators of the TLR signaling pathways. This induction subsequently results in the expression of both IL-1R-associated kinase (IRAK-M) and peroxisome proliferator-activated receptor-*γ* (PPAR), which are negative regulators of IRF7, the transcription factor that induces the expression of IFN-*α* and IFN-*β* [13]. The long-term persistence of these negative regulators impairs the clearance of MERS-CoV infections; therefore, MERS-CoV persistence at the site of infection is established [22].

MyD88-dependent and TRIF-dependent signaling both use the TLR-4 pathway to activate downstream effectors [22]. However, mice lacking TLR-4 have more severe SARS-CoV infections than wild-type mice have. Thus, the protective signaling role through TLR-4/TRIF might be considered a distinctive feature in the pathogenesis of some coronaviruses [23]. We believe that using some special adjuvants as agonists for TLR-4 and TLR-3 plus the expressed MERS-CoV-S protein may help to improve the immunogenicity against MERS-CoV infection.

**Figure 1:**
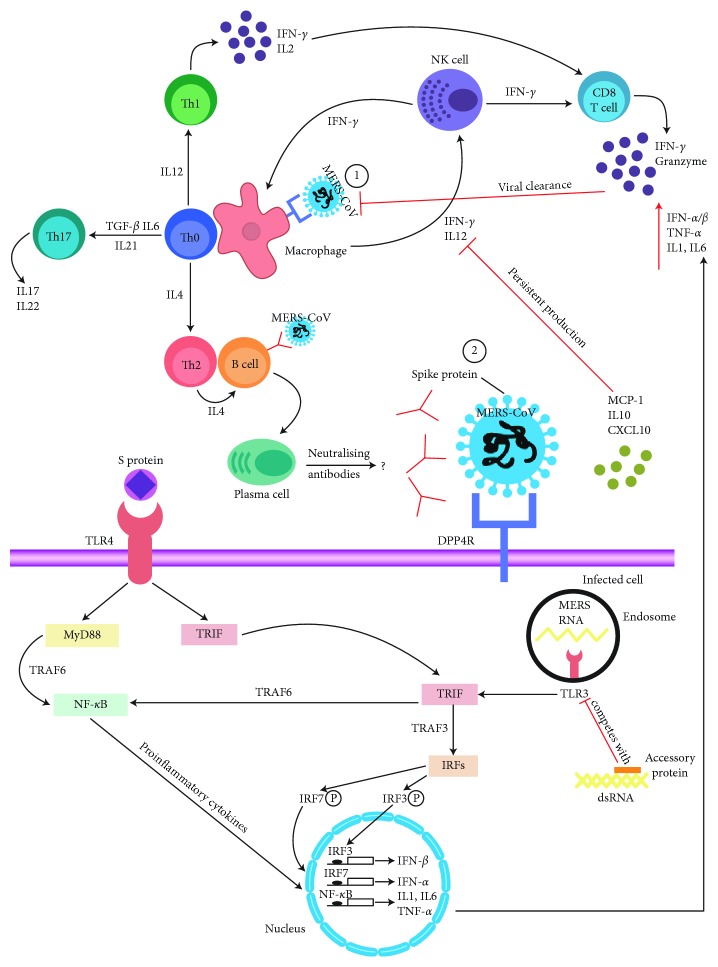
The proposed schematic representation of the immune response to MERS-CoV infection and how the invading virus is processed during an infection. (1) MERS-CoV infects macrophages through DPP4 binding, and then macrophages present MERS-CoV antigens to Th0 cells. This process leads to T cell activation and differentiation, including the production of cytokines associated with the different T cell subsets (i.e., Th1, Th2, and Th17), followed by a massive release of cytokines for immune response amplification. The continued production of these mediators due to viral persistence has a negative effect on Th0, NK, and CD8 T cell activation by inhibiting IL12 and IFN-*γ* production. However, CD8 T cells produce very effective mediators, such as IFN-*γ* and granzyme, to clear MERS-CoV. It is still unclear whether long-term or short-term protective antibodies are produced during neutralizing antibody production against MERS-CoV. (2) Attachment of MERS-CoV to DPP4 on the host cell through S protein leads to the appearance of genomic RNA in the cytoplasm. An immune response to dsRNA can be partially generated during MERS-CoV replication. TLR-3 sensitized by dsRNA and cascades of signaling pathways (IRFs and NF-*κ*B activation via TRAF3 and TRAF6, respectively) are activated to produce type I IFNs and proinflammatory cytokines. The production of type I IFNs is important to enhance the release of antiviral proteins for the protection of uninfected cells. Sometimes, accessory proteins of MERS-CoV can interfere with TLR-3 signaling and bind the dsRNA of MERS-CoV during replication to prevent TLR-3 activation and evade the immune response. TLR-4 might recognize S protein and lead to the activation of proinflammatory cytokines through the MyD88-dependent signaling pathway. Virus-cell interactions lead to strong production of immune mediators. The secretion of large quantities of chemokines and cytokines (MCP-1, IL10, and CXCL10) is promoted in infected cells in response to MERS-CoV infection. These chemokines and cytokines in turn recruit lymphocytes and leukocytes to the site of infection. Red arrows refer to inhibitory effects. Black arrows refer to activating effects.

TLR-3 initiates the activation of interferon regulatory factors (i.e., IRF7 and IRF3) after binding with its ligand in a manner independent of MyD88 [20]. The TLR-3 agonist (poly IC) has recently been reported as a potential therapy for MER-CoV infection in a mouse model [24]. After the administration of poly IC, type 1 interferon expression is induced (IFN-*β* and IFN-*α*) [25] (Figure 1). Thus, different effectors, such as natural killer cells, CD8 T cells, and macrophages, are activated, and their antiviral effects are triggered [26, 27]. It is known that the proinflammatory cytokine response (such as TNF-*α* and IL6 production) against any infection has several drawbacks in the host, such as pathological damage to tissues [28]. In contrast, the proinflammatory cytokine response can control viral dissemination. Understanding the TLR signaling pathways in the context of MERS-CoV infection will contribute to control of the viral infection, thereby mitigating the risk of its spread.

Generally, IFNs play important roles during some viral infections and can be stimulated by double-stranded RNAs (dsRNAs) [15]. A study conducted by Chu et al. in 2014 demonstrated that monocyte-derived dendritic cells (Mo-DCs) infected with MERS-CoV exhibit no expression of IFN-*β*, despite the marginally early expression of IFN-*α* [29]. However, another recent study failed to stimulate the proinflammatory innate response and failed to produce type I IFNs in vitro in cultured infected cells, primary human airway epithelial cells and Mo-DCs infected with MERS-CoV [30, 31]. The mechanisms behind this response may be initially related to interference with the NF-*κ*B signaling pathway, which is usually responsible for the induction of the proinflammatory response [32]. In addition, it is possible that the number of regulatory T cells induced at the site of infection has negative impacts on the expression of proinflammatory cytokines. Recently, treatment with IFN-*α* showed some promising trends in MERS-CoV-infected cells. The effect of the application of IFN-*α* in MERS-CoV-infected cells was 50-100-fold greater than that in SARS-CoV-infected cells [33]. Additionally, Hart et al. studied different IFN products and two different antiviral drugs, namely, ribavirin and mycophenolic acid (MPA), against MERS-CoV infection (Hu/Jordan-N3/2012) in vitro. The researchers showed that IFN-*β* has a potent inhibitory effect on MERS-CoV in comparison to that of other tested IFNs. Compared with ribavirin treatment, MPA treatment caused a stronger inhibition of viral replication in vitro, with an IC_50_ of 2.87 *μ*M [34]. MPA was shown to enhance IFN-stimulated gene expression, suggesting that MPA is dependent on the modulation of the expression of IFN-stimulated genes [35]. Thus, MPA might provide an alternative treatment for MERS-CoV infection. IFN-*β* and MPA as combination or single therapies might provide great benefit as potent inhibitors in the treatment of MERS-CoV-infected patients by reducing viral loads. The FDA has approved the use of both IFN-*β* and MPA for other indications, and these therapies are currently in use [34].

MERS-CoV infects and replicates inside macrophages and subsequently induces the expression of MHC-I, MHC-II, and costimulation-related genes [28]. Some researchers have investigated the great impact of the MERS-CoV spike glycoprotein on the responsiveness of macrophages and monocytes (THP-1 cells) via TLR-4 signaling pathways [13]. They have shown that the MERS-CoV-S protein has a negative impact on the production of proinflammatory cytokines (IL6 and TNF-*α*). In contrast, this virus increases the production of anti-inflammatory cytokines, such as IL10. As suggested by Nicholls et al., the cytokines released by alveolar macrophages could have substantial effects on the pathogenicity of SARS-CoV [36].

MERS-CoV infection stimulates the production of type I IFNs (IFN-*α* and IFN-*β*) by infected cells, which leads to the release of some chemokines, such as MCP-1, CXCL10, and the cytokine IL10, which are responsible for T cell recruitment [37, 38]. It is known that CD4 helper T cells (T_h_1) and natural killer (NK) cells require signaling by IL12 and IFN-*γ* for their activation. IFN-*γ* contributes to the activation of the two main arms of the immune response that help clear viruses: NK cells and CD8^+^ T cells [26].

However, the persistence of MCP-1 and CXCL10 has a negative impact on the expression of IL12 and IFN [37] (Figure 1). Mahallawi et al. found no remarkable differences in the IL12 and Th2 cytokine expression profiles between MERS-CoV-infected patients and a healthy control group [39]. In the context of MERS-CoV infection, the production of both CXCL10 and IL10 increased in patients' sera within 0-3 days postinfection. However, the patients who did not recover or tolerate the infection had persistent viral replication due to the increase in the expression levels of both CXCL10 and IL10 [37]. Thus, these cytokines have a negative impact on the antiviral Th1-mediated effects [40]. Another study reported the upregulation of IL10 expression in MERS-CoV-infected patients compared to that in healthy volunteers [39]. IL10 has a positive impact on the production of proinflammatory cytokines mediated by the Janus kinase/signal transducer and activator of transcription (JAK-STAT) pathway [41]. This cytokine production is positively correlated with the Th2-mediated response (i.e., IL4 and IL13 expression), which in turn inhibits the type II IFN (IFN-*γ*) expression level [42]. The expression of this cytokine is also associated with persistence in some other viral infections, such as human immunodeficiency virus (HIV), hepatitis C virus (HCV), and hepatitis B virus (HBV) [43].

## 3. MERS-CoV-Adaptive Immunity

### 3.1. MERS-CoV Cell-Mediated Response

T cells are the key players required for immunity against viral infections; CD4^+^ T cells facilitate virus-specific antibody production through the T-dependent activation of B cells. However, CD8^+^ T cells are cytotoxic and kill virus-infected cells [44]. Through the comparison of T cell-deficient BALB/c mice (mediated by the transduction of Ad5-hDPP4) with control mice and B cell-deficient mice, researchers determined that T cells are able to survive and destroy virus-infected cells in the infected lung [45]. This report may highlight the important roles of T cells but not B cells in controlling and fine-tuning the pathogenesis and outcomes of MERS-CoV infection. Zhao et al. infected Ad5-hDPP4-transduced BALB/c mice with either SARS-CoV or MERS-CoV. Subsequently, these mice were challenged with both viruses 5 weeks later. The results confirmed that the initial infection with SARS-CoV led to a significant decrease in MERS-CoV titers at day 5 postinfection. Thus, a cross-reactive T cell response may result in decreasing MERS-CoV titers [45]. The roles of T and B cell responses in the context of MERS-CoV infection were studied. Both activated CD8 cells and anti-MERS-CoV antibodies were crucial for the clearance of the initial infection and protection against a subsequent challenge with the virus, respectively. This finding implies that the response to MERS-CoV generally occurs through antibody-mediated immunity [45]. Another study demonstrated that mice vaccinated with DNA encoding the modified SARS-CoV-S glycoprotein developed protective immunity resulting from the induction of T cells and the production of neutralizing antibodies. The protection was mainly due to an antibody-dependent (and not T cell-dependent) response [46]. Yang et al. reported that specific memory cells against spike proteins have no effect on viral clearance, even 2 days postchallenge [46]. This result was confirmed when virus-specific T cells were depleted. However, this effect of cell depletion was not timely monitored at different intervals [47]. Hence, the antiviral effects of the depleted cells may be important during later infection time points, leading to the persistence of the viral infection and promoting viral survival. Moreover, during the course of MERS-CoV infection, the virus invades the immune system and downregulates MHC-I, MHC-II, and CD80/86 in antigen-presenting cells (APCs), which subsequently inhibit the T cell response [48]. These events may further impair the functions of B cells [49] and T cells via downregulation of the DPP4 receptors [29]. Recently, the induction of immunosuppression during the course of MERS-CoV infection, through promoting apoptosis of T cells, was identified as another strategy to manipulate survival pathways by the host immune response [50]. It has been thought that DPP4 may play significant roles in the signaling and activation of T cells during the course of MERS-COV infection [51]. Both CD4^+^ and CD8^+^ T cells isolated from human peripheral blood (PB), tonsils, spleens, and lymphoid organs could be infected with MERS-CoV but not with SARS-CoV. This infection pattern might be attributed to the low expression of the SARS-CoV receptor, namely, angiotensin-converting enzyme 2 (ACE2), in T cells [49]. A recent study reported that CD4^+^ helper T cells were more susceptible to MERS-CoV infection. Additionally, MERS-CoV can induce T cell apoptosis by activating both the intrinsic and extrinsic apoptosis pathways [49]. Interestingly, there was a significant upregulation in the expression level of IL17 in MERS-CoV-infected patients [39]. T helper cells, especially Th17 cells, produce the proinflammatory cytokine IL17 via the STAT3 and NF-*κ*B signaling pathways [52]. This finding suggests that MERS-CoV infection promotes the induction of Th17 cytokines. These Th17 cytokines can recruit neutrophils and monocytes to the site of infection or inflammation and lead to the activation of other downstream cytokine and chemokine cascades, such as IL1, IL6, TNF-*α*, TGF-*β*, IL8, and MCP-1 [53].

### 3.2. MERS-CoV-Antibody-Mediated Response

Neutralizing antibodies are very potent in neutralizing viral infectivity through blocking their entry into host cells. The detection of specific antibodies to MERS-CoV in human serum is considered one of the confirmative diagnoses for infection with MERS-CoV. It is crucial to determine whether these antibodies are potent arms of the adaptive response to MERS-CoV infection. However, detection of the anti-MERS-CoV antibody response occurs on days 14–21 after infection [54–56]. The antibody concentrations increase with time and last more than 18 months, and the long-term antibody response depends on the severity of the infection [57]. The anti-SARS-CoV antibody response can remain detectable for up to 24 months postinfection [58] and then begins to gradually decrease until it completely disappears 6 years after infection [59].

It is known that coronaviruses express surface spike glycoproteins, which are considered predominant antigenic proteins that stimulate the antibody response [60]. These antibodies might be used for targeting spike proteins and blocking the entry of the virus into host cells [60]. Therefore, designing monoclonal antibodies directed against these proteins is preferable for protection in contrast with vaccine preparation, which is a time-consuming and laborious process. To date, no vaccine for either SARS-CoV or MERS-CoV is available in the market despite some laboratory clinical trials.

A study reported by Coleman et al. proved that mice vaccinated with coronavirus S nanoparticle technology generate a high level of neutralizing antibodies against homologous viruses. These antibodies are not cross-protective with heterologous viruses [60]. On the other hand, a previous study performed by Chan et al. showed that antibodies recovered from the serum of some convalescent SARS patients might cross-react with MERS-CoV or neutralize it [61]. Another study suspected that these antibodies may cross-react with MERS-CoV because the epitope that is recognized by the cross-reactive antibodies might not be situated in the Spike 1 protein of SARS-CoV or at least might not be present in the receptor binding domain (RBD) [62].

The finding of anti-MERS-CoV antibodies in Kenya in 1992 [63] was consistent with the results reported recently from Saudi Arabia. These findings suggest that MERS-CoV has been circulating in dromedary camels for more than 20 years in Saudi Arabia [64]. A total of 52.2% of these antibodies that were specific to the spike protein required a high titer to neutralize MERS-CoV, with a range between 1 : 80 to 1 : 800, and only 6% had a neutralizing antibody titer (more than 1 : 800) [63].

By using a recombinant MERS-CoV spike protein subunit 1-based ELISA (rELISA) [65], it was found that the antibody against spike protein was optimal for screening. It was also determined that 29.5% of serum samples isolated from dromedary camels were positive when tested by rELISA. In addition, all positive samples were tested using an established recombinant immunofluorescence assay, which showed that 93.4% of the samples had antibodies against MERS-CoV [63].

By using an anti-MERS-CoV nucleocapsid indirect ELISA and following 34 months of infection with MERS-CoV, the neutralizing antibody titers at 34 months of infection in 86% of human serum samples were the same as those after 13 months of infection. However, 29% of patients had a lowered titer of neutralizing antibody after 34 months of infection [66]. The low titer of antibodies in a few patients is attributed to viral shedding and persistence at the site of infection (i.e., mucosal site) or might be attributed to the neutralizing effect, which leads to a reduction in the proportion of antibodies. On the other hand, the long-term persistence of antibodies in most patients might be explained by the MERS-CoV infection inducing long-lived memory B cells, which in turn form antibody-secreting plasma cells that are stored in the bone morrow until reexposure to the same virus or similar epitopes. Thus, these antibodies may protect humans from reinfection with MERS-CoV, even though the concentration of antibodies in the serum is low. Thus, we can conclude that the type of assay used should be reconsidered to have a great sensitivity for viral detection. Further testing is required to identify conserved proteins in this virus serotype to induce effective antibody-mediated immunity as well as cell-mediated immunity.

In fact, a longitudinal study carried out in dromedary camels in the United Arab Emirates (UAE) between 2014 and 2015 demonstrated that serum samples collected on the day of dam parturition had a high level of specific anti-MERS-CoV antibodies, but in calves, the antibodies were not detectable [67]. The reason for this result might be that the camel calves consumed very low amounts of colostrum during the first 24 hours and that the low levels of IgG antibodies in the dams' milk started to decline at 24 hours postparturition [68]. However, the level of specific anti-MERS-CoV antibodies in the serum from calves increased, peaked on day 7 postparturition, and then decreased during the next 6 months; thus, neutralizing activity was functionally lost in 50% of the calves, and the rest had low antibody titers [67]. This result may explain the survival of MERS-CoV in these calves, and serum IgG antibodies may not be sufficient for protective immunity. Despite potential neutralizing antibodies in the serum, anti-MERS-CoV antibodies might be used as a valuable indication for viral diagnosis. Thus, a serum sample that is positive for specific antibodies would serve as a confirmative diagnosis of MERS-CoV infection.

## 4. MERS-CoV Vaccines

In this section, we summarize the most recent findings with respect to MERS-CoV vaccine development, particularly vector- and RBD-based vaccines.

### 4.1. MERS-CoV Viral Vector-Based Vaccine

Recombinant-modified vaccinia virus Ankara (MVA) expressing the full-length MERS-CoV spike protein induced a high-level specific neutralizing antibody response in vaccinated BALB/c mice via intramuscular (i.m.) [69, 70] or subcutaneous (s.c.) routes of injection [70]. Several studies have reported that the i.m., s.c., and intradermal routes (i.d.) used for vaccine administration provide a good level of protection against both MERS-CoV and SARS-CoV infections. This protection level may be attributed to the downstream stimulation of a favorable immune response [71, 72]. However, this type of vaccine may elicit antibody-mediated disease enhancement (ADE) by the nonneutralizing epitopes encoded by the S glycoprotein [73]. MVA-MERS-CoV-S-specific neutralizing antibody titers were highly detectable after either a single immunization (day 21) or booster immunization (day 40) with a dose of 10^7^ or 10^8^ plaque forming units (PFUs) [70]. Compared to the antibody levels in previous studies of SARS-CoV [74], these high antibody levels were efficient in blocking the epitopes of MERS-CoV spike protein. The confirmation of antibody specificity was carried out by testing the serum obtained after the second booster against SARS-CoV; however, the serum showed undetectable levels of neutralizing antibody to SARS-CoV [69]. Previously, a correlation between the levels of specific neutralizing antibodies to the spike protein and the protectiveness of immunization in animals infected with SARS-CoV was shown [74, 75]. Thus, MVA-MERS-S vaccination can effectively stimulate humoral and cell-mediated responses. Additionally, the vaccine efficiency was similar in a study that conducted clinical testing, especially with respect to the immunogenicity of other recombinant MVA vaccines [76, 77]. MVA-MERS-CoV-S elicited a specific IFN-*γ*-producing CD8^+^ T cell response against MERS-CoV infection by both the i.m. and s.c. routes following a prime-and-boost immunization regime. The specific CD8^+^ T cells from the mouse spleen were stimulated with the MERS-CoV-S291 peptide and showed an upregulation in IFN-*γ* expression. The booster vaccination increased the level of the MERS-S291-specific CD8^+^ T cell response by 3-fold [70]. Another study using an adenovirus type 5- (Ad5-) based vaccine expressing MERS-CoV-S proteins demonstrated the ability of this vaccine to induce systemic and mucosal antigen-specific immunity when administered via the i.m. or intragastric (i.g.) route [78]. This study proved that the sera of the vaccinated mice had high levels of antigen-specific IgGs and neutralizing antibodies, but no specific T cell response was detected in the case of the vaccines administered through the i.g. route. However, immunization through the i.m. route generated persistent antigen-specific T cell responses in both the spleen and lungs of the vaccinated animals [78]. Protective neutralizing antibodies and T cell-mediated responses were strongly elicited after challenging monkeys immunized with an adenoviral-based SARS vaccine expressing S1, M, and NP proteins with SARS-CoV [79]. Additionally, the elicitation of both humoral and cell-mediated responses has been confirmed with an adenoviral-based SARS-CoV vaccine encoding RBD [80].

On the other hand, the preexisting immune response against the MVA and adenovirus vectors is one of the limitations of using viral vector-based vaccines, which may cause harmful immune responses and inflammation [69, 81, 82]. Although the MVA-MERS-CoV-S vector is a strong inducer of both cellular and antibody responses, there are some concerns about the safety of using these vector-based vaccines.

### 4.2. MERS-CoV-RBD-Based Vaccine

Some studies have shown that the RBD-based subunit of the SARS-CoV vaccine is very effective and safer than the viral vector candidates [83, 84]. As reported, immunization of mice with an RBD-based vaccine by the i.m. route induces long-term protection against SARS-CoV infection [85]. Thus, targeting MERS-CoV-RBD protein-1 is one of the strategies for vaccine development [86]. The immunogenicity of this fragment within the MERS-CoV-S spike protein was tested and evaluated. Remarkably, the MERS-CoV-S377-588-Fc has stronger immunogenicity than the other MERS-CoV-RBD proteins (S367-388-Fc, S358-588-Fc, and S367-606-Fc) and elicits significantly higher titers of neutralizing antibodies in vaccinated mice [87]. These antibodies are capable of blocking the binding of MERS-CoV-RBD to its receptors. This is a promising trend in the development of effective and safe MERS-CoV vaccines [88, 89]. Two available antibodies (REGN3051 and REGN3048) were capable of binding the RBD of the S protein and inhibiting its interaction with DPP4. Therefore, a potential inhibitor was developed [90]. These antibodies were tested in a mouse model and were at least effective at inhibiting MERS-CoV replication [91], but further testing of these vaccine candidates in dromedary camels should be conducted. Moreover, a developed humanized monoclonal antibody (mAb YS110) against DPP4 was reported and demonstrated inhibition of MERS-CoV infections [91]. Another study revealed that both mice and rabbits develop high titers of neutralizing antibodies when stimulated with 377-588-Fc [87]. Intranasal (i.n.) vaccination with a MERS-CoV-RBD-based subunit vaccine has a strong potential to induce a mucosal neutralizing IgA response against the RBD and MERS-CoV S proteins [87].

The 358-588 RBD fragment was shown to induce neutralizing antibodies in immunized rabbits [89], whereas fragment 377-662 was effective in immunized mice [88]. These results demonstrated that the expression of the recombinant S377-662-Fc protein in the RBD vaccine potentially triggers the production of specific antibodies in mice through the s.c. route after two booster vaccinations; these neutralizing antibodies are effective against MERS-CoV in Vero E6 cells in vitro [88]. Because this pathogen is mucosal-dependent, the administration of an i.n. vaccine that stimulates a potent mucosal IgA response would be a better route for the induction of an increased mucosal immune response to prevent infection with MERS-CoV. It has been reported that both local and systemic immunity are induced effectively by the i.n. immunization pathway. The mucosal IgA in vaccinated animals can provide cross-protection against homologous and heterologous strains of influenza virus and lead to long-term protection due to the memory response [92]. Moreover, long-term protection was reported in SARS-CoV infection by the i.n. route [80]. Zhang et al. reported some promising results using S377-662-Fc protein for mouse immunization via the i.n. route [93]. In contrast to the s.c. pathway, mice immunized intranasally with S377-662-Fc protein exhibited markedly high levels of specific IgA in the lungs [93]. However, compared with the sera from mice treated by the s.c. route, the sera of mice immunized through the i.n. route contained slightly higher levels of mucosal IgA [94]. On the other hand, the levels of IgG in mice immunized with S377-662-Fc protein via both s.c. and i.n. routes were the same. After a single dose administered via the i.n. route, the level of IgG was relatively low [94]. After several doses over 6 months, the titers of MERS-CoV-S1-specific IgG were high and persisted long term. These data confirm that the MERS-CoV-S377-662-Fc protein is capable of triggering a strong local mucosal response, especially by the i.n. route [93]. Moreover, a specific cell-mediated immune response in the spleen of immunized mice was generated by MERS-CoV-S377-662-Fc protein [94]. This finding indicates that both mucosal humoral and cellular immune responses might contribute to MERS-CoV prevention once induced by the RBD subunit vaccine. Due to the ability of the MERS-CoV-RBD-based vaccine to induce effective systematic and mucosal neutralizing antibodies, this subunit might be considered a promising potential candidate for the prevention of MERS-CoV infection [93]. A strong specific IgG antibody response against RBD was generated by Ad-MERS-S 4 to 16 weeks postimmunization. The levels of induced antibodies were significantly higher after immunization via the i.m. route than after immunization via the i.g. route. This result implies that this recombinant virus is capable of inducing a long-term specific antibody response via both routes [78].

### 4.3. The Potential Uses of Adjuvants in Association with MERS-CoV Vaccines

The neutralizing antibody production caused by most coronaviruses usually increases when immunization is used in conjunction with an adjuvant. It was reported that the inoculation of mice with MERS-CoV spike protein alone did not induce sufficient antibody production unless the viral protein was bound to an adjuvant, which then caused a potent response of neutralizing antibodies [60]. Both alum and MF59 adjuvants can elicit antigen-specific antibodies and cellular-mediated responses [95] and might be used for MERS-CoV subunit vaccine administration. However, alum alone cannot induce a potent Th1 response unless combined with another adjuvant, such as glucopyranosyl lipid A (a synthetic TLR-4 agonist). This cocktail will improve the effectiveness of the MERS-CoV-RBD-based subunit vaccines [96]. Coleman et al. showed that the immune responses to both SARS-CoV and MERS-CoV-S nanoparticles were increased significantly by approximately 15- and 7-fold by using the adjuvants alum and MF59, respectively [60]. This study was consistent with another investigation in terms of the production of anti-SARS-CoV neutralizing antibodies in mice [97]. Matrix M1 consists of two different components of saponin fractions: Matrix-A (Fraction-C saponin), which is the weaker part of saponin, and Matrix-C (Fraction-C saponin), which is a highly active adjuvant [98]. In clinical trials, Matrix M1 was proven to be a potent adjuvant [99]. In another study, using Matrix M1 as an adjuvant significantly boosted the level of antibody titers by 68-fold. [60]. The utilization of adjuvants might enhance immunogenicity and safety in MERS-CoV vaccine development.

## 5. Future Prospects

The innate immune response is an important element of antiviral defense and adaptive immunity. Further investigation is required to achieve a better understanding of the innate immune response to MERS-CoV. Thus, having sufficient data on highly pathogenic MERS-CoV, including understanding the mediators of innate immunity, their pathways, and how this virus can be regulated, will pave the way to develop effective antiviral therapeutics and vaccine candidates. To provoke a specific immune response without disease progression, an effective vaccine should be formulated. To date, there is no effective vaccine targeting the specific protein antigens of MERS-CoV.

MERS-CoV contains other accessory viral proteins (M, ORF 4a, ORF 4b, and ORF 5) [100]. In addition to the MERS-CoV-S protein, the membrane (matrix) protein and other structural proteins may have important roles in the development of other vaccine candidates. Thus, targeting these viral proteins might facilitate vaccine development [73] by limiting their ability to inhibit IFN production through binding to the dsRNA of the virus [100–102]. Additionally, the MERS-CoV-RBD-based vaccine, particularly the specific residues 377-606, induced strong and high antibody titers that were shown to have a neutralizing effect against MERS-CoV infection in immunized animals. However, testing these residues in human cells is required to confirm their efficacy as a potential vaccine. Thus, a protein-based vaccine that contains RBD should have great potential to elicit a highly neutralizing antibody response against several epitopes [73]. Additionally, as MERS-CoV targets the mucosa of the respiratory tract, designing a vaccine that enhances the induction of strong immunity via the i.n. route would be one of the best strategies to block MERS-CoV infection. Several factors affect the immunogenicity of vaccines. Each vaccine has an appropriate route of administration. Thus, selection of the optimal route of administration and the proper adjuvant with a specific, conserved antigen will play significant roles in MERS-CoV vaccine development and the efficacy of these candidate vaccines.
